# Medical Professors in Dental Colleges

**Published:** 1867-11

**Authors:** 


### EDITORIAL DEPARTMENT.

*Medical Professors in Dental Colleges*—Much idle clamour has been raised about medical professors in Dental Colleges, and some who favor certain institutions consider it a matter deserving of special commendation that they are officered exclusively by Dentists. The direct bearing of this fact upon their competency as instructors is not easy to perceive.

If this objection to medical professors rests upon any other foundation than a petty and unworthy jealousy, it must pre-suppose some fancied incapacity of a medical man to give proper instruction to dental students. Now this incapacity must depend either upon the nature of the studies themselves or the training of the physician who undertakes to direct them.

The former hypothesis will scarcely be conceded to be correct by those who are aware of the constant, persistent, and at length successful efforts of dentists to get their profession recognized as a speciality of medicine. It unquestionably requires a certain amount of strictly medical knowledge to prosecute it with success, and it is therefore perfectly right that it should be so regarded. There is therefore, nothing whatever in the nature of the studies to prevent a physician from being a suitable man to aid a dental student in acquiring a knowledge of his profession; unless it be supposed that a thorough study of an entire science disqualifies one for teaching a part of it. Upon this hypothesis, a professor could not be trusted to teach a boy algebra because he was thoroughly versed in mathematics, from the first four rules of arithmetic to the highest generalizations of the calculus.

What better reason then, have we for supposing that a medical man's training disqualifies him for the duties in question, for this is the only other supposable case? The chairs occupied by Doctors of Medicine in Dental Colleges are Therapeutics, Anatomy and Chemistry. Let us see how far a physician may be, from the fact that he is a graduate in physic, supposed incompetent to communicate information in those sciences. Chemistry, to begin with, is a general science of great scope and innumerable applications. It is notorious that many Medical schools have chosen for professors of this science gentlemen who never studied Medicine, for the simple reason that they were the most competent teachers they could find. If then, in the wider field of general medical science, professors may be chosen who are not M.D's, why need the narrow range of dental surgery require a D.D.S. to teach its pupils? What is needed here is a general knowledge of chemistry, together with its special applications to the science and art of dentistry. Surely no one is better qualified to impart the former information than he who has made the science of chemistry a special study. As for the latter, any chemist, worthy of the name, ought, upon a very short notice, to get a complete knowledge of what is needed.

Anatomy, too, is a science of generalities, as well as specialities. Surely a man is not less capable of teaching the structure of the head and neck, because he has acquainted himself with that of the entire system. A thorough knowledge of all the nerves is not inimical to a particular acquaintance with the ramifications and connections of the fifth pair.

Can any thing more be said against the selection of a physician to teach Therapeutics? Here again the student wants general knolwedge. He desires some insight into the general principles of the healing art. Now shall he take, for this purpose, a dentist whose daily practice deals with only a few of the agents of the Materia Medica; or shall he select a physician who is constantly called upon to use them all, and who therefore has a far more extensive and practical knowledge of their virtues, their indications and their contra-indications? The statement of the question is its answer.

The truth is that the sole qualifications required in a teacher of any art or science, should be a thorough familiarity with what he professes to teach, and a good faculty of imparting the knowledge he possesses. Any other demands are idle, absurd and utterly irrelevant.

